# The Low Carb Program for people with type 2 diabetes and pre-diabetes: a mixed methods feasibility study of signposting from general practice

**DOI:** 10.3399/BJGPO.2021.0137

**Published:** 2021-12-15

**Authors:** Emma Scott, Mishkat Shehata, Arjun Panesar, Charlotte Summers, Jeremy Dale

**Affiliations:** 1 Warwick Medical School, Coventry, UK; 2 DDM Health, Coventry, UK

**Keywords:** Diabetes mellitus, type 2, Health promotion, Primary health care, Diet, carbohydrate-restricted, Feasibility study, General practice, prediabetes

## Abstract

**Background:**

Evidence shows type 2 diabetes mellitus (T2DM) can be effectively treated with a reduced-carbohydrate diet to support weight loss. Digital apps are increasingly used to support weight loss, yet little is known about their use as part of general practice diabetes care.

**Aim:**

Determine the feasibility of signposting from routine NHS general practice to a digital weight management tool (Low Carb Program) for patients with T2DM and pre-diabetes.

**Design & setting:**

Mixed-methods feasibility study implemented within routine general practice consultations at four practices in the Midlands, England.

**Method:**

General practices offered signposting to eligible patients attending consultations of any type during a 4-week recruitment period. Rates of offering and accepting signposting were recorded, with program registration, program completion, and self-reported health outcomes (weight, haemoglobin A1C [HbA1c]).

**Results:**

Signposting was offered to 351 patients; 160 (45.6%) accepted, 103 (29.3%) registered with the intervention and 43 (26.9% of patients accepting signposting) completed the programme. GPs reported that signposting added between 1–4 minutes to the consultation length. Patients completing the programme reported greater weight loss (7.2kg versus 1.6kg, *P*<0.001) and HbA1c improvements (-9.1mmol/mol versus 1.7mmol/mol, *P*<0.001) compared to those who did not, and were more likely to reduce the number of prescribed diabetes medications in general practice.

**Conclusions:**

Signposting from real-world general practice to the *Low Carb* *Program* is feasible and can potentially improve diabetes outcomes. Further research should explore whether the process of signposting can be enhanced to increase registration, identify whether additional practice-led support leads to increased programme completion, and confirm the intervention’s clinical and cost-effectiveness.

## How this fits in

The feasibility of signposting patients with T2DM to digital health interventions from primary care services has not been extensively examined. This research demonstrates the feasibility of referring patients with T2DM from primary care consultations to digital services that support weight loss. It provides insight into how signposting can benefit patients, and supports the need for more in-depth research to establish the cost and clinical effectiveness of such signposting.

## Introduction

T2DM affects approximately 4.2 million adults in the UK.^1^ Obesity is the primary risk factor for the onset and progression of T2DM, and 80% of people with T2DM are overweight or obese.^2,3^ Effective, accessible, and scalable weight management approaches that can be delivered as part of routine primary care are urgently needed.

Obesity is an important risk factor. Losing weight provides significant health benefits and losing excess body weight contributes to reduction in the risk of T2DM, heart disease, and sleep apnoea.^4,5^

The benefits of a low-carbohydrate diet (<130g carbohydrate per day) in weight management are increasingly recognised. Recent meta-analyses comparing low-carbohydrate and low-fat diets found a significantly greater reduction in body weight for the low-carbohydrate group.^3,6^ A recent systematic review and meta-analysis of research evaluating low-carbohydrate diets (<130g/day or <26% of a 2000 kcal/day diet) and very low-carbohydrate diets (<10% calories from carbohydrates) for at least 12-weeks in adults with T2DM reported moderate-to-low certainty evidence that patients adhering to a low-carbohydrate diet for 6 months may experience T2DM remission without adverse consequences.^7–9^

Integrating digital technology into primary care has the potential to increase care access, improve patient outcomes, and reduce costs. Smartphone apps may augment the reach of health services through remote self-management and behavioural change.^10^ The challenge is how to facilitate signposting patients to these digital health tools in a time-efficient manner that does not increase the burden on already stretched primary care services. Scalable access to the resources that educate and support patients with pre-diabetes and T2DM to make long-term dietary changes are needed. Digital weight management interventions are available, but adoption and engagement varies.^11–15^

### Objective

The study’s aim was to determine the feasibility of signposting patients with T2DM and pre-diabetes during routine NHS general practice consultations to the '*Low Carb Program* *'* *,* a digital weight management app.

Specific objectives included determining healthcare professionals’ (HCPs’) willingness to opportunistically signpost patients; acceptance of signposting and app registration rates; intervention uptake and participant retention; and exploring experiences of intervention use with surveys and interviews.

### Intervention

The '*Low Carb Program* *'* is an NHS-approved app that supports people with T2DM and pre-diabetes to make dietary and lifestyle changes to achieve weight loss. It provides evidence-based structured education and goal-focused behaviour change coaching to support weight loss through adoption of a low-carbohydrate diet (130g carbohydrate per day).^16^ Modules unlock weekly over 12-weeks, delivered using videos, ranging from 3–12 minutes, and written content (see Table 1 for syllabus). Participants make behavioural changes based on goals at the end of each module. The app engages the Social Ecological Model of Health^17–20^ and solution-focused coaching, placing the focus on a person’s present and future circumstances and goals. Patients are supported with self-monitoring tools (HbA1c, weight, food diary); and recipes recommended by artificial intelligence to match patients’ dietary preferences and based on liked recipes, foods logged in their food diary, and recipes liked by other members who share similar demographics.

**Table 1.** 'Low Carb Program' syllabus

Week	Topic	Objective
1	Welcome to the type 2 diabetes / pre-diabetes program	Safety notes and alerts to medications that require healthcare professional teams' assistance Benefits of a reduced carbohydrate diet for people with type 2 diabetes Benefits of a reduced carbohydrate diet for people with pre-diabetes
2	Type 2 diabetes and diet	Factors that affect blood glucose levels Encouragement to engage with their healthcare providers
3	Controlling portion sizes	Introducing visual methods for interpreting portion size
4	Real versus processed foods	Identifying and eliminating refined and processed food
5	Healthy and unhealthy fats	Discussion of fat types and making appropriate choices depending on goals
6	Vegetables	Demonstrating the carbohydrate content of vegetables and cooking methods
7	Fruit	Reviewing the amount of sugar and starch in fruit and vegetables
8	Snacks and desserts	Examining low-carb snack, dessert, and drink options
9	Drinks	Tips on alcohol and eating out options
10	Eating out and takeaways	Managing eating on the go and when travelling Making healthier takeaway and food choices
11	Practical ways to eat less carbohydrates	Practical tips for reducing carbohydrate intake further Safety information — highlighting medications that require healthcare professional team assistance
12	Timing your meal	Introducing the principles of reducing the eating window using the 16:8 model

## Method

### Study design, practice participation, and participant recruitment

This study was a mixed methods feasibility study with the intervention implemented within routine general practice consultations.

Four general practices were recruited, aiming for diversity in setting and size. Practice staff, including GPs, nurses, and healthcare assistants (HCAs), received: a 10-minute slide presentation about the intervention; a booklet explaining the low-carbohydrate approach and what app use involves; referral cards to enable free patient access; a brief signposting statement to use with eligible patients (Appendix 1); and a deprescribing protocol.^21^ (Appendix 2).

Each practice was asked to implement its own approach to incorporate signposting into the consultation, utilising a signposting statement that drew upon Prospect Theory^22^ and the ‘*Ask*’ domain of motivational interviewing^23^ to give a gain-framed message that highlighted the intervention’s positive benefits,^24^ and incorporated GP endorsement to encourage uptake.^25^

An electronic pop-up was installed on the practices’ patient management system to trigger a signposting prompt when eligible patients presented. It offered various response options: accepted signposting, declined, inappropriate to offer.

Patients aged ≥18 years, with a confirmed diagnosis of T2DM or pre-diabetes and a body mass index ≥25 kg/m^2^, who presented for any reason during the recruitment window were eligible for signposting if the consulting HCP felt it appropriate. Patients who accepted signposting were given a referral card to gain free access to the intervention. Participant information sheets and digital consent forms were presented on a study-specific '*Low Carb Program'* webpage. A 4-week recruitment period was anticipated as sufficient to allow at least 300 patients to be offered signposting.

Practice staff (GPs, nurses, and HCAs) delivering care to patients with T2DM and pre-diabetes on a regular basis at the participating practices were invited to use the signposting resources.

### Data collection

Participating practices provided information on T2DM and pre-diabetes rates and pop-up prompt responses using population report searches, including age, sex, and ethnicity of all participants offered signposting. The 43 ethnic identities for patients with a diagnosis of T2DM were collapsed into five overarching ethnic groups for analysis: White, Asian, Black, Mixed, or unknown/missing. Data were anonymised and non-identifiable.

Self-reported patient registration (age, sex, height, ethnicity, diabetes type, and duration), module completion, and health data were extracted from the intervention. Patients reported their weight, HbA1c, and diabetes medications at baseline and regular 3-monthly intervals.

Behavioural change interventions rarely achieve 100% adherence.^26^ Participants completing ≥75% (≥9/12) modules were classified as intervention completers. Participants completing between 2 and 8 modules classified partial completers, and those completing ≤1 modules as non-completers. Retention at 3 and 6 months was defined as providing self-monitoring data at these time points.

Participants registered with the intervention were invited to complete a survey (13 closed questions and three open-text questions) about their experience of the signposting and using the app, 3 and 6 months post-registration.

Practice staff participated in either a one-on-one interview or focus group.

### Data analysis

Quantitative analysis was undertaken using SPSS (version 26). Comparison of patient demographics at different stages of participation was undertaken using 2 and Student *t-*tests. Exploratory outcomes assessments were undertaken using Student *t-*tests, Pearson Correlation Coefficients, and analyses of variance (ANOVAs). Interviews and focus groups were audio-recorded and transcribed verbatim prior to analysis. Framework Analysis^27^ of the data was undertaken using a framework developed from the interview questions.

## Results

### Recruitment and retention

Participating practices covered a total population of 65 118, ranging in size from 8200 to 26 500 patients, in urban, suburban, and rural communities. Mean T2DM incidence rate across the practices was 4.7% (range 2.2%, 7.2% of the practice list) and for pre-diabetes 3.7% (range 1.9%, 4.9% of the practice list).

**Figure 1.**
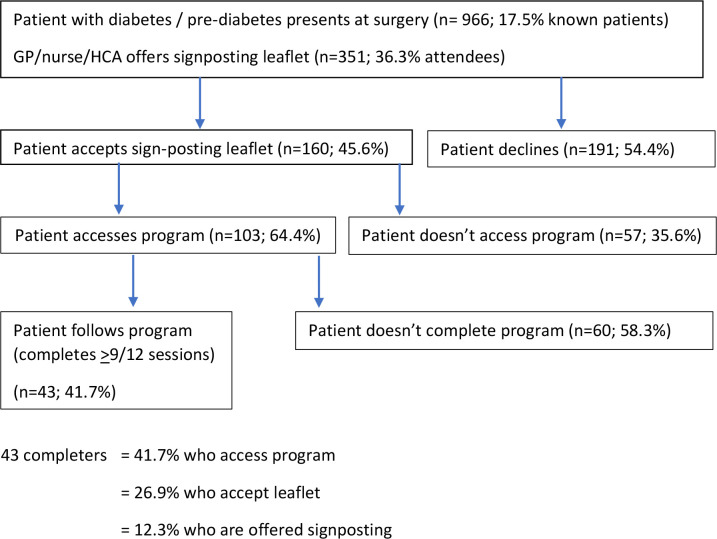
Flow of patients through the study

Figure 1 shows the patient recruitment pathway from signposting to completion of the intervention. Across the practices, 966 eligible patients presented during the 4-week recruitment window; 351 (36.3%) patients were offered signposting; of which 160 (45.6%) accepted a referral for the app (practice range 21.0–65.0%) and 103 (29.3%) registered with the intervention (Table 2).

**Table 2.** Signposting acceptance rates by practice

	Practice A	Practice B	Practice C	Practice D	Total
Eligible patients during 4 week recruitment period	141	585	63	177	966
Offered signposting	60 (42.6%)	190 (32.5%)	63 (100%)	38 (21.5%)	351 (36.3%)
Accepted signposting	39 (65.0%)^a^	89 (46.8%)	24 (38.1%)	8 (21.1%)^b^	160 (45.6%)

Significant difference in acceptance rate by practice (χ²=19.9; *P*<0.001). ^a^Higher than expected. ^b^Lower than expected.

Forty-three patients (41.7% registrants; 26.9% of patients accepting signposting) completed the intervention (≥9/12 modules) and 34 (33.0% of registrants) partially completed the programme (2–8 modules).

### Participant characteristics

There was a difference in signposting acceptance by ethnicity (χ² = 30.64, *P*<0.001), with a higher acceptance rate among Black patients (Table 3). After accepting signposting, White patients were more likely to register (χ^2^ = 47.0, *P*<0.001).

**Table 3.** Patient demographics

	Offered signposting	Accepted	Declined	Registered with LCP
Mean age, years (±SD)	63.6±12.8	59.9±12.3^a^	66.6±12.4^a^	57.5±12.9
Sex (male)	53.3%	55.6%	51.3%	46.6%
Ethnicity:				
White	106	40	66	75^b^
Black	41	28^a^	13	9
Asian	32	14	18	19^b^
Mixed	83	16	67^a^	0
Unknown^c^	14	7	7	0
Missing	75	55	20	0
Diagnosis:				
T2DM				86 (83.5%)
Pre-diabetes				11 (10.7%)
Not stated				6 (5.8%)
Time since diagnosis:				
<5 years				40 (38.8%)
5–10 years				36 (35.0%)
11–15 years				10 (9.7%)
16–20 years				11 (10.7%)
>20 years				6 (5.8%)

LCP = Low Carb Program.

a Significant difference between those who accepted and declined the signposting offer (*P*<0.05).

b Number registering appears greater than number accepting, likely due to participants with unknown or missing data in practice self-reporting ethnicity during the registration process.

c Coded as unknown on patient management system in practice.

Patients accepting signposting were younger than patients who declined (mean age 59.9±12.2 years versus 66.6±12.4 years; *t* = 5.1, *P*<0.001). Similarly, patients who registered with the programme were younger than the overall cohort (57.5±13.0 years versus 64.9±12.6 years; *t* = 5.5, *P*<0.001). There was no sex difference between patients accepting and declining signposting, nor between those who registered and those who were eligible for signposting.

### Registration with the intervention

Of the 103 patients registered, 46.6% were male, mean age 57.5±12.9 years, and 72.8% White, 18.4% Asian and 8.7% black. Eighty-six (83.5%) had a diagnosis of T2DM, 11 (10.7%) had pre-diabetes, and six (5.8%) identified as obese but did not indicate diabetes diagnosis. Patient T2DM or pre-diabetes diagnosis date ranged from within the last 5 years (38.8%) to >20 years ago (5.8%; Table 3). The mean baseline weight of those who registered was 96.4±19.0 kg (Table 4).

**Table 4.** Self-reported outcome measures, by number of *Low Carb Program* modules completed

	Number of modules completed						
	All participants	9–12 modules		2–8 modules		1 or no modules	
*N*	103	43 (41.7%)		34 (33%)		26 (25.2%)	
	Baseline	Baseline	Follow up	Baseline	Follow up	Baseline	Follow up
Mean weight, kg	94.4±19.0	107.3±19.2^a^	100.1±16.8^b,c^	88.2±12.4	87.3±12.3^b,c^	89.2±17.4	None of these participants provided follow up data for comparison
Weight range, kg	59.9–156.0	69–156	63–148	59.9–110.0	60.4–109.0	64.0–142.6	
HbA1c, mmol/mol	66.1±23.4	70.5±28.2	61.4±24.0^b^	65.6±23.9	63.9±23.8^d^	60.3±10.8	
HbA1c range, mmol/mol	42.1–149.2	44.3–149.2	42.1–129.5	42.1–140.4	42.1–118.6	42.1–82.5	
Median number of medications taken	2	2	1^b^	2	1	2	
Range number of medications taken	0–3	0–3	0–3	0–3	0–3	0–3	

^a^Significant difference compared to both other groups at baseline (*P*<0.05). ^b^Significant difference within group from baseline (*P*<0.001). ^c^Significant difference between groups at follow up (*P*<0.001). ^d^Significant difference within group from baseline (*P*>0.01)*.*

Of 103 registrants, 43 (41.7%) completed the intervention (>9/12 modules), 34 (33.0%) partially completed the programme (2–8 modules), and the remaining 26 (25.2%) were non-completers.

### Outcome data

Seventy-eight patients (75.7%) were retained and provided post-intervention follow-up data at 3-months and 60 (58.3%) at 6-months from baseline. At the most recent data collection (7.3±2.9 months from baseline), mean weight loss was 7.2±5.0 kg for completers and 1.6±1.5 kg for partial completers (*t* *=* 6.1, *P*<0.001).

Fifty-one patients provided follow-up HbA1c data (Table 4), which was a drop from 65.2±23.4 mmol/mol at baseline to 62.5±23.7 mmol/mol at follow-up (*t* *=* 5.6, *P* *<0*.001). Participants with a higher HbA1c tended to complete the programme, and for these individuals there was a mean reduction in HbA1c of 9.1 mmol/mol. Changes in weight and HbA1c were correlated (*r* = 0.393, *P* = 0.004).

At baseline, patients reported a median of two (range 0–3) prescribed medications for diabetes. Eighteen (41.8%) completers and seven (20.6%) partial completers reduced medications.

### HCP views

Nineteen GPs, practice nurses, and HCAs participated in interviews or focus groups prior to initiating signposting. Eleven follow-up interviews were completed post-signposting.

Most were positive about signposting to the intervention using the materials provided and the potential for positive patient outcomes.

Main concerns were lack of time to opportunistically signpost within a 10-minute consultation, the need to tailor signposting to individual patients’ requirements, and uncertainty about patient acceptance. Provider-specific barriers included confidence in deprescribing medication and its consequential workload.

They reported that some patients needed additional assistance with registering with the app, but most were happy to proceed independently and liked the app being accessible on-demand. HCPs who were more enthusiastic about promoting lifestyle change reported greater success at signposting patients and had higher acceptance rates. Most felt they would continue to signpost to the intervention.

The time involved in signposting varied across the practices. Two practices reported signposting added 2–4 minutes to consultations, depending on information requested by patients. One practice offered signposting in a flu clinic and found it was possible to signpost in under a minute. Medication changes, where required, were estimated as adding up to 10 minutes more. More follow-up with patients was required to adjust diabetes management following weight loss. However, the short-term increase in consultation time or frequency was expected to be outweighed by longer-term reduction in appointments as diabetes control improved.

### Patient experience

Nineteen (18.4%) patients responded to the 3- and 6-month questionnaires. Responders were broadly representative of the participants registered with the programme, although male participants were slightly overrepresented.

Most (*n* = 17, 89.5%) felt that the GP consultation was a good time to be offered the *Low Carb Program* referral card; all were pleased to have been offered signposting, and most (*n* = 18, 94.7%) found it easy to understand what the intervention entailed.

## Discussion

### Summary

This study demonstrates the feasibility of signposting patients with T2DM or pre-diabetes to the *Low Carb Program* as part of routine general practice care. With a minimal implementation package, and a pragmatic approach to delivery designed to avoid disruption to normal general practice workflows, the study demonstrated rates of signposting acceptability to eligible patients (45.6%); uptake (accessing the programme; 64.4%), and adherence (completing the programme; 41.7%). Patients who completed ≥9 of the 12 intervention modules reported an average weight loss of 7.2 kg compared to 1.6 kg for partial completers. Similarly, patients who completed the programme reduced HbA1c by 9.1 mmol/mol (0.83%) compared to 1.7 mmol/mol (0.16%) for partial completers, and about one third of participants reduced the number of diabetes medications that they were taking.

Primary care teams welcomed their patients gaining free access to the app, appreciated the guidance that they received, and experienced the intervention as adding about 2–4 minutes time to standard care. The practice with the lowest rates for offering signposting also had the lowest rate for patient acceptance; factors that affect the extent to which patients are offered and accept signposting need further consideration. Although the completion of follow-up surveys by patients was low, those who did complete surveys expressed high intervention satisfaction.

### Strengths and limitations

A strength of this study is its pragmatic, real-world design. The protocol allowed practices to implement signposting to the intervention in whichever way worked best within their existing workflows. Through a mixed methods design, data were available from general practice record systems; the *Low Carb Program*; patient surveys; and HCP interviews and focus groups, which allowed a detailed understanding of the intervention’s implementation, use, and effect. Another strength is the recruitment of practices in varied settings with diverse populations, so increasing the generalisability of this study’s findings.

Participating HCPs found the implementation materials helpful but there is scope to develop these to support more effective programme signposting and patient follow-up. As a pragmatic study, it was not prescriptive about what GPs should do after signposting, but greater patient follow-up might have encouraged an increased level of intervention registration.

Use of self-reported data is a limitation, although previous research has found that self-reported health outcomes are close to actual values.^28,29^ Although beyond the scope of this feasibility study, future research should extract data on HbA1c, weight, and medications directly from GP record systems rather from than patients’ self-report data. It is difficult to rule out the effect of medication on weight and HbA1c status. Age, sex, ethnicity, and time with diabetes may have been confounding variables. However, analysis of these variables fell outside of the scope of assessing the feasibility of signposting to the intervention in general practice.

Challenges identified in this study were minimal with most attributable to lack of time. In busy primary care practices, it is difficult to recruit staff to maximise patient recruitment and interview all providers pre- and post-recruitment. Incentivisation should be considered in future research. Another limitation is the signposting statement, which could be made clearer and more inviting. Further research should explore signposting statements that will be more likely to engage potential participants.

Survey responses disproportionately reflected experiences of participants who had completed the programme, hence their enthusiasm should be interpreted cautiously.

### Comparison with existing literature

These findings are comparable with those of similar interventions. HeLP-Diabetes, a web-based tool^30^ reported lower uptake, engagement, and completion rates (9.4% of those who registered). Evaluation of a UK-based digital lifestyle intervention reported a similar mean weight loss at 6-months: 7.12 kg (-7.50%; SD 6.37; *P*<0.001)^31^ compared to 7.2±5.0 kg in the present study. A randomised controlled trial evaluating the effectiveness of a digital intervention in Australia found a small improvement in HbA1c in the intervention arm compared to baseline of similar scale to that observed in the present study.^32^

Based on a ‘light-touch’ 10-minute presentation to staff and variable usage of materials prompting a 2–4-minute brief intervention, this intervention was able to achieve high patient uptake within the context of primary care. Other interventions typically require staff resources. HeLP-Diabetes used practice staff to identify eligible patients from medical record searches and recruited using consultations and SMS.^30^ Given the feasibility of signposting to the intervention in primary care without burdening staff, it is prudent to research which benefits can be widely replicated.

### Implications for research and practice

The NHS Weight Management Programme is a government initiative launching in the UK in late 2021, with general practice the primary source of patient referrals.^33,34^ The programme will incentivise HCPs to signpost patients with obesity to a digital app following a brief consultation delivered through general practice. The results of this feasibility study are particularly relevant and transferable to this initiative, as there is minimal research on the feasibility of signposting patients to digital apps. These results demonstrate that signposting to a digital app is feasible as part of routine general practice care, and the high signposting rate of demonstrates HCPs’ willingness to opportunistically signpost patients without financial incentives.

This study demonstrates the feasibility of signposting patients with T2DM and pre-diabetes to the *Low Carb Program*. With the implementation of this very brief intervention as part of routine care, about 12% of eligible patients complete the programme; it also has high acceptability to primary care teams and patients. Further research should test this finding with more practices and patients; refine implementation guidance; and enhance engagement strategies to maximise programme take-up, completion, and patient benefits.
